# Successful ultrathin bronchoscopy with cryobiopsy for diagnosing and removing mucus plugs in allergic bronchopulmonary mycosis mimicking lung cancer

**DOI:** 10.1002/rcr2.1359

**Published:** 2024-04-23

**Authors:** Yuki Takigawa, Hiromi Watanabe, Ken Sato, Mayu Goda, Tomoyoshi Inoue, Miho Fujiwara, Suzuka Matsuoka, Kenichiro Kudo, Akiko Sato, Keiichi Fujiwara, Takuo Shibayama

**Affiliations:** ^1^ Department of Respiratory Medicine NHO Okayama Medical Center Okayama Japan

**Keywords:** allergic bronchopulmonary mycosis, cryobiopsy, mucus plug, ultrathin bronchoscope

## Abstract

In patients presenting with abnormal pulmonary nodules, especially those with a history of asthma, allergic bronchopulmonary mycosis should be considered. Eosinophil counts and IgE levels should be checked in such patients.

## Graphical abstract

We present a case of successful ultrathin bronchoscopy with cryobiopsy for diagnosing and removing mucus plugs in a patient with allergic bronchopulmonary mycosis mimicking lung cancer.

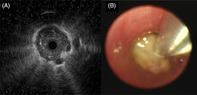

## CLINICAL IMAGE

**FIGURE 1:**
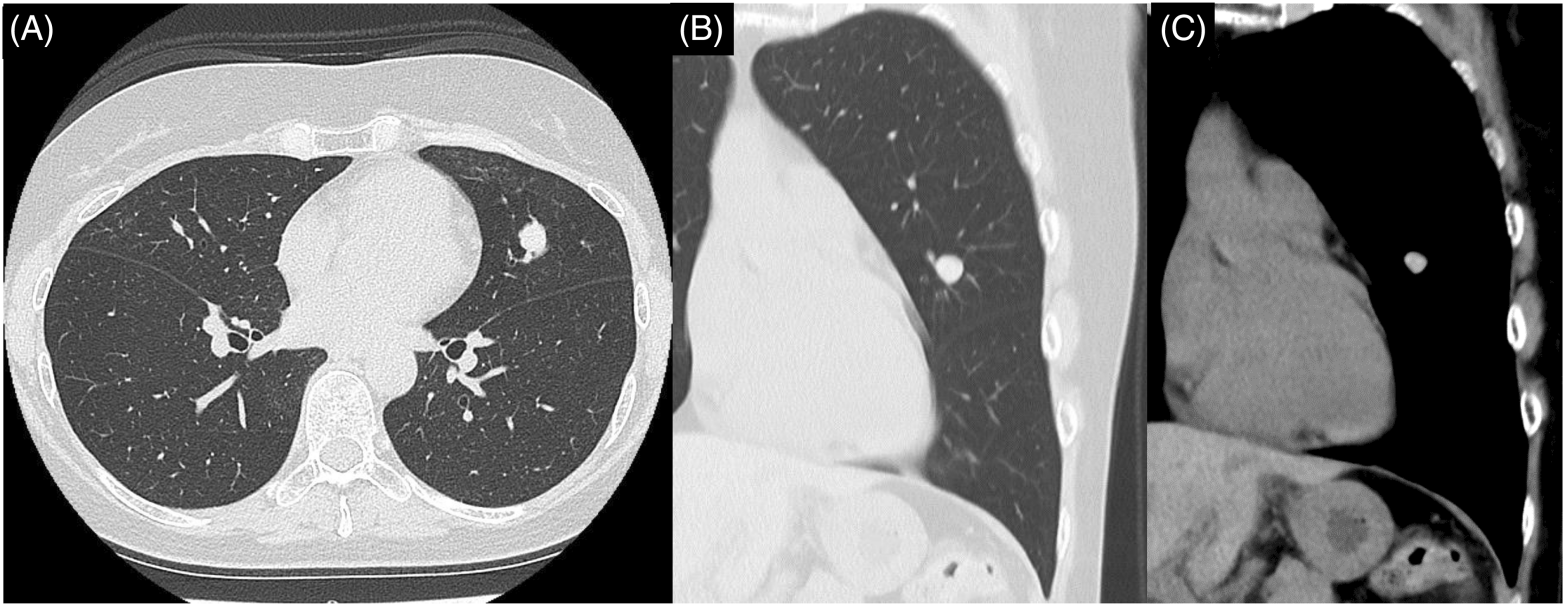
Chest computed tomography in a 53‐year‐old woman with asthma revealed a solid nodule (84 HU) with a maximum diameter of 11 mm in the S5 of the left lung. (A: axial, B: coronal lung window and C: mediastinal window).

**FIGURE 2:**
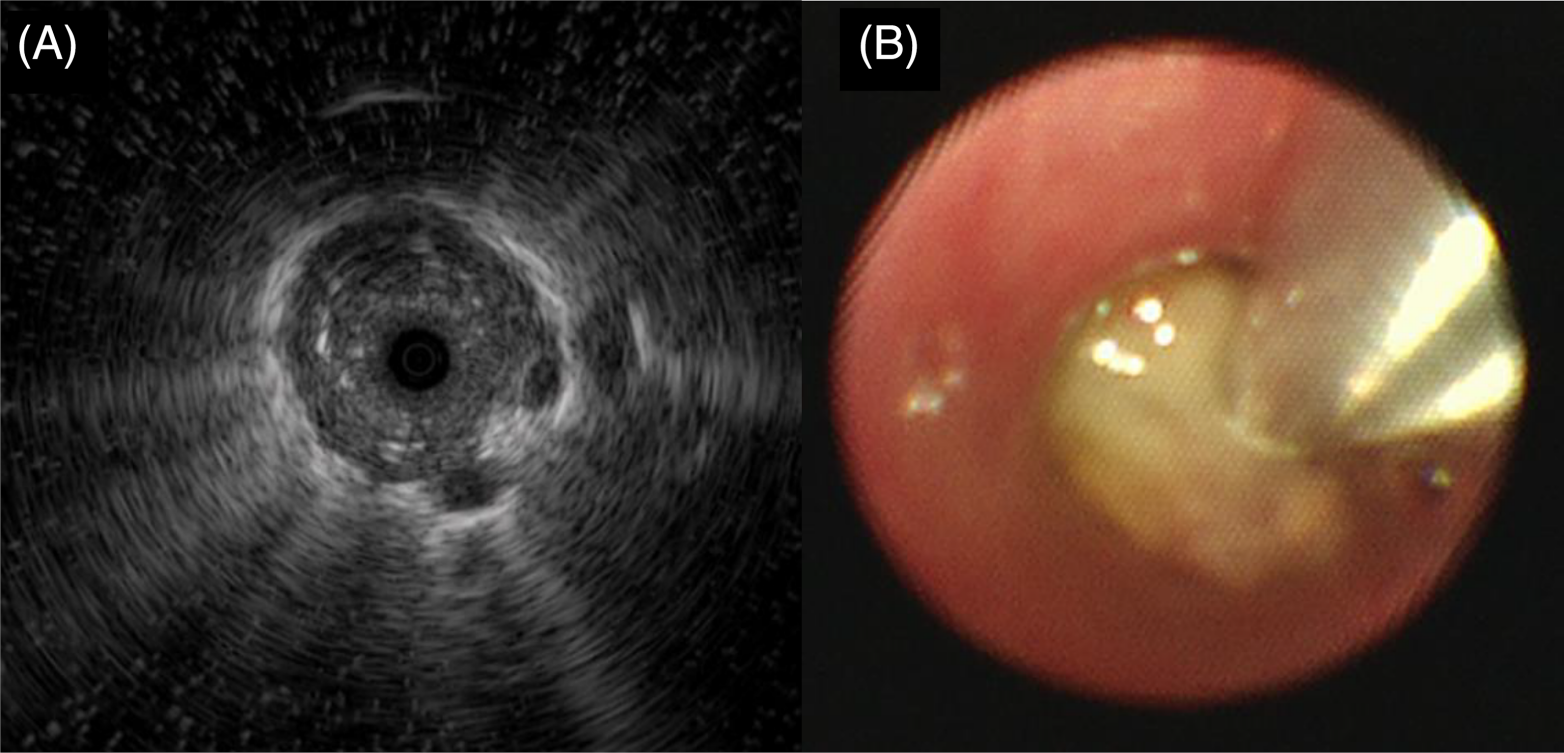
An ultrathin bronchoscope was advanced into the left B^5^ bronchi. A radial endobronchial ultrasound probe confirmed within the lesion (A). The bronchoscopy image in a well‐inhaled state revealed a yellowish tumour, indicating a mucus plug (B). These findings led to the diagnosis of allergic bronchopulmonary mycosis.

**FIGURE 3:**
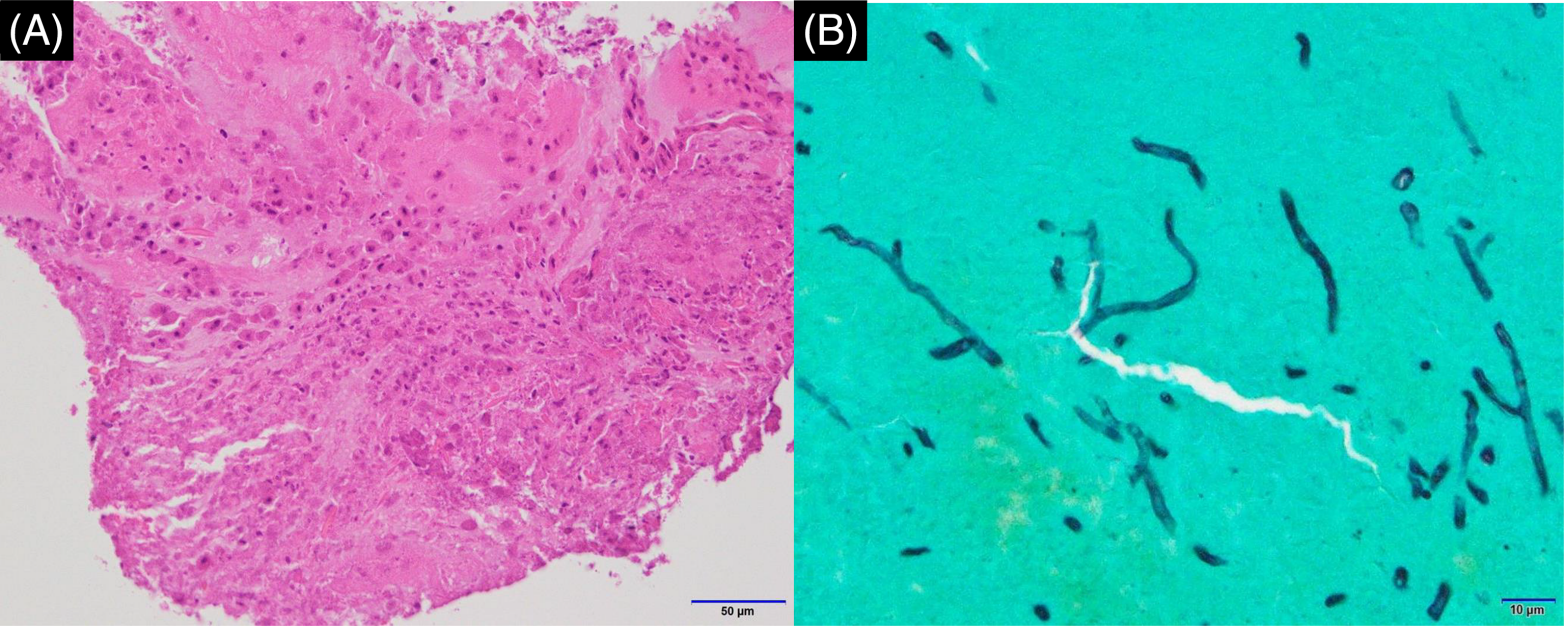
Histopathological findings of the tissue obtained by cryobiopsy show Charcot–Leiden crystals with eosinophils (A). Grocott staining shows Y‐shaped fungal hyphae (B).

**FIGURE 4:**
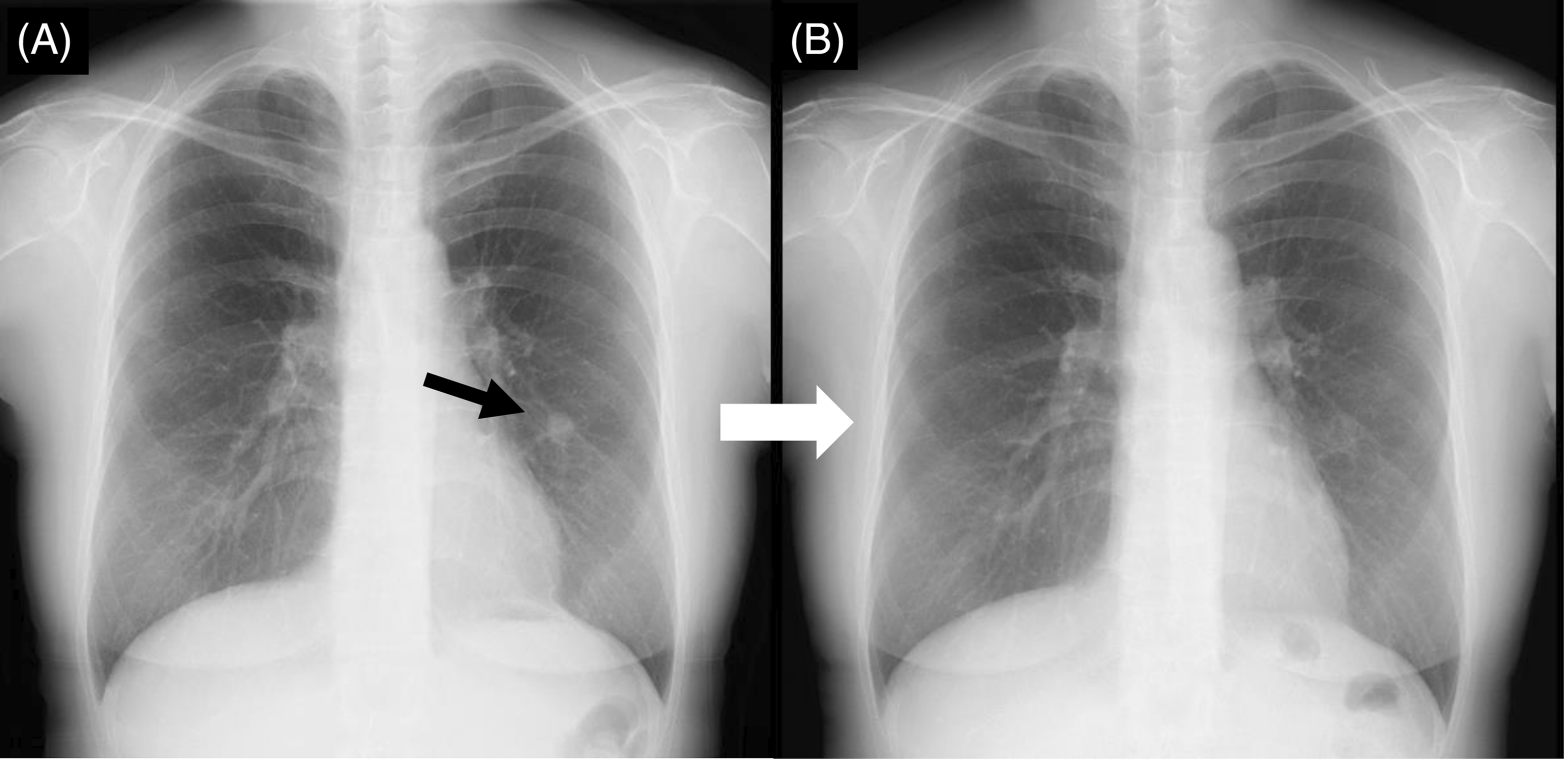
Radiographic imaging showed the disappearance of the nodule (black arrow) 3 months after bronchoscopic removal.(A: Chest x‐ray on admission and B: 3 month after bronchoscopic removal).

**FIGURE 5:**
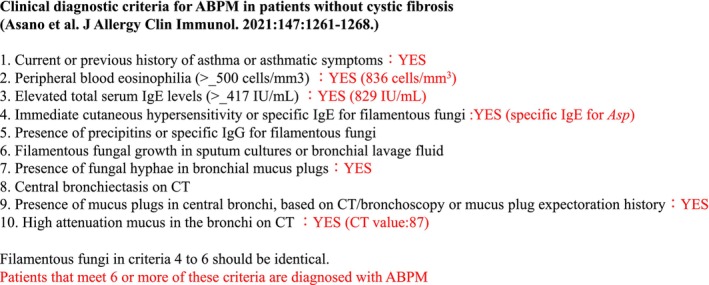
Clinical diagnostic criteria for allergic bronchopulmonary mycosis in the present patient.

A 53‐year‐old woman with a history of asthma presented to our hospital for examination of a nodular pulmonary lesion. Chest computed tomography revealed a solid nodule with a maximum diameter of 11 mm in the S5 of the left lung (Figure 1). Bronchoscopy was performed with an ultrathin bronchoscope, which was wedged in a sub‐segmental branch of B^5^. The radial endobronchial ultrasound probe confirmed within the nodule. The nodule was very soft, and sufficient tissue could not be obtained by forceps biopsy (Figure 2). Therefore, we performed additional cryobiopsy. The tissue obtained by cryobiopsy was considered a mucus plug, which was removed using the cryoprobe to prevent airway inflammation at the early stage of allergic bronchopulmonary mycosis (ABPM).^1^ Histopathological findings showed eosinophils and Charcot–Leyden crystals, and Grocott staining revealed Y‐shaped fungal hyphae (Figure 3). Radiography confirmed the bronchoscopic removal of the nodule (Figure 4). *Aspergillus*‐specific IgE levels were found to be elevated, meeting the diagnostic criteria for ABPM^2^ (Figure 5). Bronchoscopy has a combined diagnostic and therapeutic role. Ultrathin bronchoscope and ultrathin‐cryobiopsy added value to the procedure because, combining them, we could reach peripheral bronchi and completely remove the mucus plug.

## AUTHOR CONTRIBUTIONS

Yuki Takigawa and Hiromi Watanabe wrote the manuscript, which was then reviewed by all co‐authors.

## CONFLICT OF INTEREST STATEMENT

None declared.

## ETHICS STATEMENT

The authors declare that appropriate written informed consent was obtained for the publication of this manuscript and accompanying images.

## Data Availability

The data that support the findings of this study are available from the corresponding author upon reasonable request.
